# Assessment of physical function of hospitalized older patients in routine clinical practice predicts 1-year mortality; A cohort study of 5,062 medical and surgical patients

**DOI:** 10.1007/s41999-026-01439-5

**Published:** 2026-02-28

**Authors:** Morten Tange Kristensen, Camilla Kampp Zilmer, Durita Viderø Gunnarsson, Theresa Bieler, Christian Hedelund Arens, Rasmus Gregersen Mottlau, Hanne Nygaard, Mette Aadahl, Charlotte Suetta, Christian Have Dall

**Affiliations:** 1 Department of Physical and Occupational Therapy, Copenhagen University Hospital, Bispebjerg and Frederiksberg, Copenhagen, Denmark; 2 Department of Clinical Medicine, University of Copenhagen, Copenhagen, Denmark; 3 Department of Emergency Medicine, Copenhagen University Hospital - Bispebjerg and Frederiksberg, Copenhagen, Denmark; 4 Center for Clinical Research and Prevention, Copenhagen University Hospital - Bispebjerg and Frederiksberg, Copenhagen, Denmark; 5 Geriatric Research Unit, Department of Geriatric and Palliative Medicine, Copenhagen University Hospital - Bispebjerg and Frederiksberg, Copenhagen, Denmark; 6 Copenhagen Center for Clinical Age Research, CopenAge, University of Copenhagen, Copenhagen, Denmark

**Keywords:** Physical capacity, Frailty, Survival, Muscle strength

## Abstract

**Methods:**

Adult patients referred for physical function evaluation by physio- or occupational therapists were evaluated with the Cumulated Ambulation Score (CAS) for basic mobility, 30sec-Sit-To-Stand-test (30s-STS) for muscle strength and function in lower extremities, and handgrip strength (HGS) as a proxy for general muscle strength. Results were categorized as normal (mean+/-1SD) or reduced (<-1SD) according to Danish sex- and age-decade reference values. The day of the first CAS assessment was used as an index for 1-year mortality in Cox regression analysis adjusted for age, sex, and multimorbidity (M3-index >1 point).

**Results:**

A total of 5062 unselected patients with a mean±SD age of 74.5±13.9 years (54% women), were evaluated with CAS in an acute (*n* = 337), surgical (*n* = 1671) or medical (*n* = 3054) ward. Of these, the 30s-STS was assessed in 85% (*n* = 4309) and HGS in 51% (*n* = 2580) patients, respectively. One-year mortality was 19.3%. The adjusted hazard ratio for 1-year mortality in patients with reduced CAS-mobility (57%), reduced 30s-STS (93%), and reduced HGS (44%) was 1.98 (95%CI, 1.71–2.29), 3.57 (2.21–5.78), and 2.06 (1.66–2.56), respectively, versus those with a normal physical function in respective tests. Correspondingly, hazard ratios for patients ≥85 years of age (n = 1185) were 2.02, 3.34 and 1.98.

**Conclusions:**

In older medical and surgical hospitalised patients with reduced physical function (CAS, 30s-STS or HGS) 1-year mortality was markedly increased. This simple test battery is rapid and low-cost and can easily be implemented in other hospitals, acute care facilities, and across sectors.

**Graphical Abstract:**

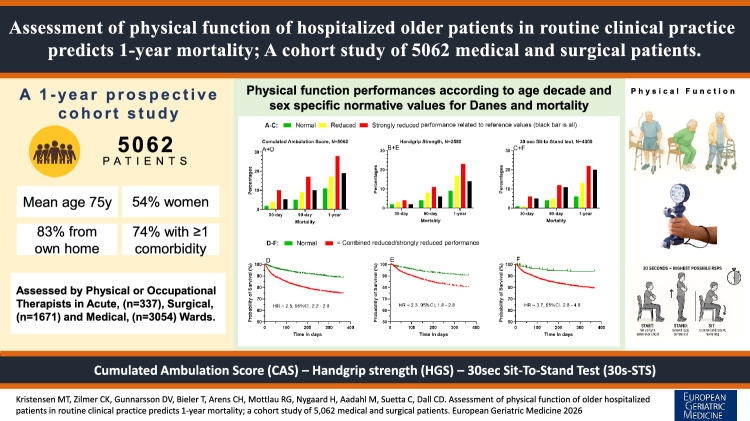

**Supplementary Information:**

The online version contains supplementary material available at 10.1007/s41999-026-01439-5.

## Introduction

Assessment of physical function is important when planning and monitoring the outcome of rehabilitation and for the identification of patients in need of further examination, i.e., patients at risk of sarcopenia [1, 2]. Reduced physical function is associated with mobility limitations, adverse health outcomes, readmission, and mortality [3–6]. Addressing frailty in ageing requires timely identification of at-risk patients to enable early intervention. This applies to all age groups and not only to older adults or to those with an already strongly reduced physical functional level or sarcopenia. Normative age-decade and sex-specific reference values for adults in Denmark have previously been described for the Cumulated Ambulation Score (CAS) for the assessment of basic mobility, handgrip strength (HGS) as a proxy for general muscle strength, and the 30s-Sit-To-Stand test (30s-STS) for specific muscle strength and function in lower extremities [7], based on data provided from previous studies [8–12]. However, to our knowledge systematic assessment of physical function in routine clinical practice using these three validated performance measures in unselected medical and surgical patients across adult age groups, hospital wards, and diagnoses has never been evaluated in an acute care hospital.

The purpose of the present study was to describe the level of physical function according to Danish reference values among adult hospitalized patients, and to examine the association between level of physical function and 30-day, 90-day, and 1-year mortality.

## Patients and methods

### Study design, setting, and population

We conducted a prospective observational cohort study at Copenhagen University Hospital, Bispebjerg and Frederiksberg (BFH), from May 2022 through April 2023, following implementation of physical function assessments as part of routine clinical practice [13]. BFH is an acute-care community hospital located in Copenhagen, serving a catchment area of approximately 483,000 inhabitants [14]. The hospital provides medical treatment and care across a wide range of specialties, including pulmonary and infectious diseases, endocrinology, cardiology, dermatology, neurology, geriatrics and palliative medicine, pulmonary, gastroenterology, and acute medicine. Additionally, BFH offers a range of acute and elective surgical services within the fields of abdominal and orthopedic surgery.

Adult patients considered in need of having their functional ability evaluated by the medical or nursing staff at the different wards and thereby referred for physical function evaluation by physio- or occupational therapists were eligible for the study. Inclusion criteria were age ≥18 years and being evaluated with at least one of the three tests of physical function: The CAS, HGS, and 30s-STS in the emergency, surgical- or medical wards. Reporting of the study follows the STROBE guideline for observational studies [15].

## Outcome

All-cause mortality verified by the Danish Civil Registration System [16] from the first objective in-hospital CAS assessment recorded in the electronic patient journal records and 1-year forward was used to record the 30-day, 90-day, and 1-year mortality.

## Exposure variables

### Cumulated ambulation score

The CAS evaluates basic mobility defined by the three activities: getting in and out of bed, sitting to standing to sitting from a chair with armrests, and walking with or without an assistive device. A score of 0-2 is used for each activity, with 6 points indicating an independent indoor ambulatory level [17]. The CAS has proven reliable in patients with hip fracture [18, 19] and i.e., valid in monitoring mobility in geriatric patients [20], and associated with; physical activity in medical patients [21], discharge destination in patients with osteoporotic vertebral fractures [22], mortality in patients with hip fractures [23], and pulmonary complications in acute high risk abdominal patients [24]. At present, the CAS is freely available in 19 languages across the world, and with a Thai version under development. The standardized CAS manual in English is available as an appendix in Jérôme et al. [19].

## Handgrip strength

For the HGS, the patients were seated in a chair with armrests with the elbow at 90 degrees and the highest value in kilograms was assessed with the Baseline BIMS Digital hand dynamometer using the dominant hand. The Baseline BIMS has proven valid in comparison with a Jamar digital dynamometer with testing following a standardized manual with three attempts and strong verbal encouragement [25]. If the third attempt was highest, more were given, but with a maximum of five attempts. If the dominant hand was injured, the other hand was used. The standardized HGS testing manual used in the present study is available as Appendix 1 in Rolsted SK et al. [25]. HGS has been proposed as a vital sign of health [26], proven reliable in acute older medical patients [27], associated with lower limb strength [28]**,** transferrin saturation after hip fracture [29] and falls [30] and i.e., used in the EWGSOP2 sarcopenia algorithm [2].

## 30s-sit-to-stand test

For the 30s-STS, the number of repetitions standing up from a chair (seat height 44-47cm) with arms crossed over the chest in 30 seconds followed a standardized manual [25]. The 30s-STS estimates lower extremity muscle strength and functional performance [31], has proven reliable in older adults [32, 33] and being associated with readmissions [34].

## Traffic light system

A traffic light system (green, yellow, and red) was applied for classification of physical function. CAS = 6 was considered a normal level (green), CAS 4-5 and 0-3 were defined as reduced (yellow) and strongly reduced (red), respectively, based on evidence and consensus [7]. Likewise, for categorization of HGS and 30s-STS performances, we used Danish age-decade and sex-specific norm data, where a normal level (green) is defined as the average ±1SD, a reduced level (yellow) as -2 – -1SD, and a strongly reduced level (red) as <-2SD [7] (Appendix 1).

The traffic light algorithm for normative reference values was integrated into the electronic patient journal system, enabling physiotherapists, occupational therapists, and other health-care professionals to obtain an automatic classification of the three test results when entering raw data into the system.

## Timeline for use of the three tests

The CAS was obtained as a pre-admission score (questionnaire-based) and again objectively at the first and last time a patient was seen for the assessment of physical function/ mobilization with a physical or occupational therapist. Correspondingly, the 30s-STS was assessed at the first and last contact, while HGS was only assessed once, and preferably early after referral to therapists. The first CAS and 30s-STS together with the HGS were used for analysis of association with mortality. Please see the timeline for all assessments in Appendix 2.

## Covariates

Age, sex, and the M3 multimorbidity index (used to describe the morbidity of the cohort) were used as covariates. The M3 Multimorbidity Index (developed in New Zealand) categorizes morbidities of patients based on ICD-10 codes for 61 chronic conditions and is considered a better predictor of 1-year mortality among the general population compared to the Charlson and Elixhauser [35].

## Other variables

To further describe the 1-year cohort, data for height, weight, residential- and smoking status were collected from the patient’s medical records.

## Statistical analysis

Patient characteristics and outcomes were presented as mean (SD) if normally distributed (based on Q-Q plots), as median with (25–75% quartiles) if not, or as numbers (percentages) if categorical.

For the crude and adjusted mortality analysis CAS, HGS, and 30s-STS performances were dichotomized into normal (green) versus a combined reduced and strongly reduced (yellow/red) level, respectively, while the multimorbidity M3-score was dichotomized as 0-1 versus >1 [35].

Crude and multivariable Cox proportional hazards models with 95% confidence intervals (CIs), adjusted for age, sex and multimorbidity variables (M3-index), were used to examine the relationship between each of the three tests of physical function and the 30-day, 90-day, and 1-year mortality. Kaplan-Meier survival graphs were used to further visualize and compare the associations between physical function and 1-year mortality. Log-minus-log versus log of survival time graph models showed parallel and independent curves for the normal and reduced performance groups for all three tests. Corresponding, multivariable Cox regression models were made for patients 85 years or older for 1-year mortality to examine if findings were like those of the entire cohort.

The level of significance was set at p < 0.05. SPSS version 29.0 (IBM Corp, Armonk, BY, USA) and GraphPad Prism (version 10.4.1, San Diego, CA, USA) software were used for the statistical analyses and figures, SAS Enterprise Guide (version 8.4) for data management.

## Ethics

Access to patient journals was approved by the local ethical committee (WZ-23016039). Data storage on a secure platform was approved by the regional data protection agency in Copenhagen (p-2023-14225). According to Danish legislation, informed consent from each included individual was not needed.

## Results

### Population characteristics

**Fig. 1:**
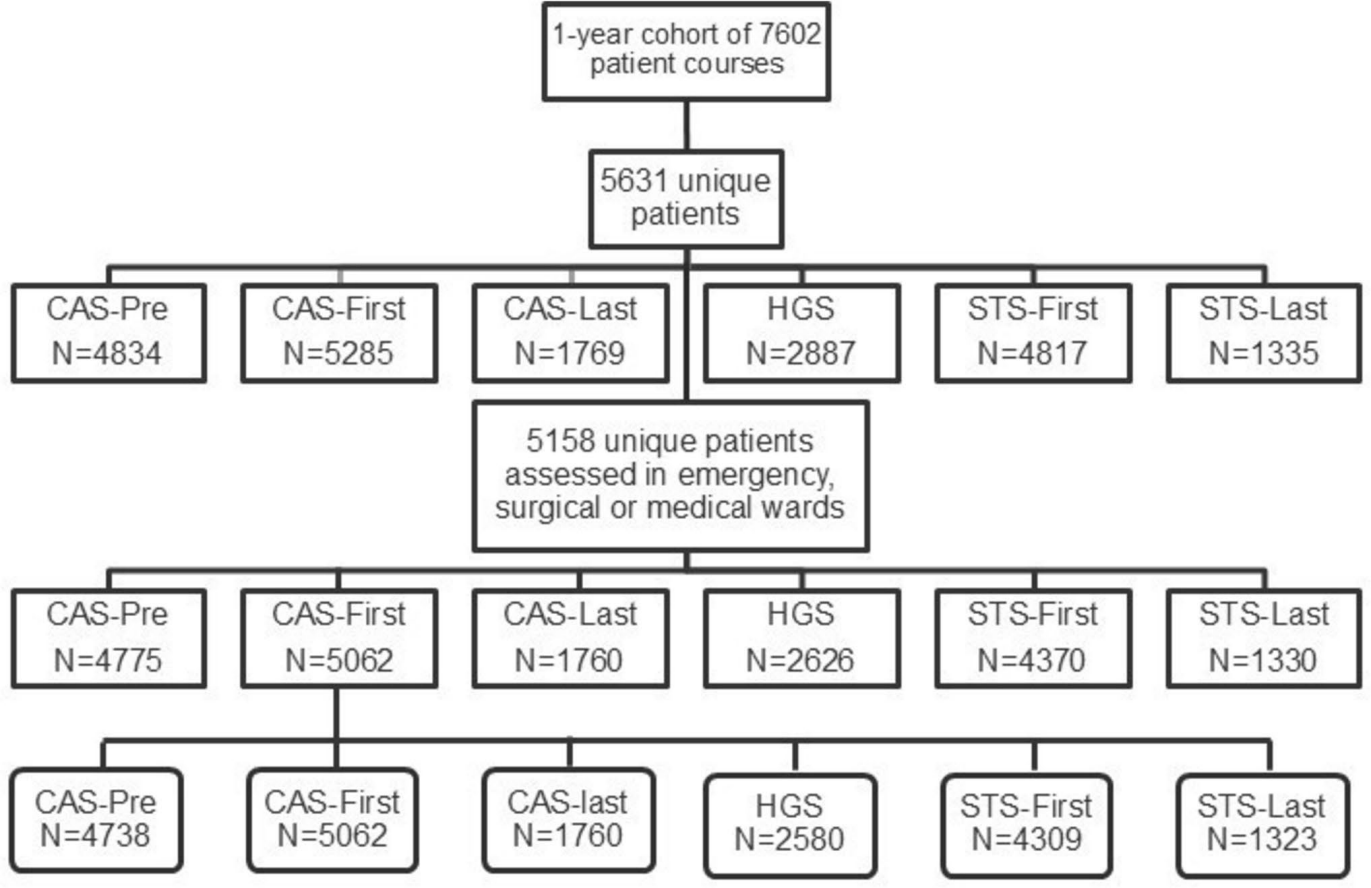
Flow chart of patient courses being evaluated with one or more of the three tests of physical function and unique patients being included in the 1-year cohort

Due to some repeated presentations over the 1-year study period, we included 7602 hospital contacts by 5631 unique patients that were evaluated by physical or occupational therapists with one or more of the three tests (Fig. 1). Among these patients, 5158 (mean (SD) age of 74.4 (14.0) years, 54% women) were evaluated in the emergency, surgical, and medical wards (Fig. 1). Differences in completeness varied largely, with most patients being evaluated with the CAS at the first objective physical- or occupational therapy assessment (*N* = 5062) and followed by the pre-admission CAS. Among the other assessments, the first 30s-STS assessment was reported for 4370 patients and followed by the HGS (*n* = 2626) as shown in the flowchart (Fig. 1). As a CAS-first objective assessment was defined as a prerequisite for being included in the up to 1-year mortality analysis, the further results are based on these 5062 unique patients being evaluated in the emergency (*n* = 337), surgical (*n* = 1671) and medical (*n* = 3054) wards (Fig. 1, Table 1), and with a reduced number of patients for HGS (*n* = 2580) and 30s-STS (*n* = 4309) assessments. Patients in the emergency and medical wards were significantly (*p*<0.001) older, had more multimorbidity (M3-index>1) and were more often men compared to patients in surgical wards.

**Table 1:** Patient characteristics and physical function performances of the 1-year hospital cohort, *N* = 5062

	All, *N* = 5062	Emergency, *n* = 337	Surgical, *n* = 1671	Medical, *n* = 3054
Age, years, mean (SD), range	74.5 (13.9), 18-102	77.4 (11.7)	71.9 (14.9)	75.6 (13.4)
Women, n (%)	2753 (54.4)	174 (51.6)	1032 (61.8)	1547 (50.7)
Height, cm, mean (SD), n = 4719	169.3 (10.3)	168.2 (10.8)	169.8 (10.3)	169.1 (10.3)
Weight, kg, mean (SD), n = 4950	72.6 (18.7)	69.9 (16.7)	75.0 (18.7)	71.5 (18.2)
Body Mass Index, mean (SD), n = 4715	25.3 (6.0s)	24.9 (8.5)	25.9 (6.0)	25.7 (5.9)
Residential status, n (%):				
-Own home	4189 (82.8)	277 (82.2)	1370 (82.0)	2542 (83.2)
-24-hour care facility (Nursing home included)	558 (11.0)	33 (9.8)	136 (4.5)	389 (12.7)
-Other	65 (1.3)	< 5	16 (1.0))	46 (1.5)
Missing	250 (4.9)	24 (7.5)	149 (8.9)	77 (2.5)
Smoking status, n (%):				
Smoker	984 (19.4)	57 (16.9)	311 (18.6)	616 (20.2)
Former smoker	1741 (34.4)	124 (36.8)	608 (36.4)	1009 (33.0)
Never	1418 (28.0)	70 (20.8)	576 (34.4)	772 (25.3)
Unknown (Missing)	919 (18.2)	86 (25.5)	176 (10.5)	657 (21.5)
M3, comorbidity level; 0/0− <1/1− <2/2+, %	25.9/48.1/19.1/6.9	18.1/48.7/24.6/6.8/1.6	32.1/48.6/15.0/3.5/0.8	23.1/47.9/20.8/6.3/2.0
Physical function tests, median (IQR), range:				
CAS (*pre-admission), 0–6 points, n = 4775	6 (6–6), 0–6	6 (6–6), 0–6	6 (6–6), 0–6	6 (6–6), 0–6
CAS (^#^first contact), 0–6 points, n = 5062	5 (3–6), 0–6	6 (5–6), 0–6	4 (3–6), 0–6	5 (3–6), 0–6
CAS (^§^last contact), 0–6 points, n = 1760	5 (3–6), 0–6	6 (5–6), 4–6	4 (3–6), 0–6	5 (3–6), 0–6
Handgrip strength, dominant hand, Kg, mean (SD), range:				
-Women, *n* = 1404	19.4 (7.4), 1.2–51.7	17.4 (6.0)	21.6 (7.5)	17.4 (6.7)
-Men, *n* = 1176	32.6 (12.1), 1.9–75.6	26.3 (9.2)	37.4 (12.6)	30.0 (10.9)
30s-STS (^#^First contact), repetitions median (IQR), range:				
-Women, *n* = 2351	0 (0–0), 0–25	0 (0–4), 0–17	0 (0–0), 0–19	0 (0–1), 0–25
-Men, *n* = 1958	0 (0–4), 0–30	0 (0–4.75), 0–20	0 (0–0), 0–19	0 (0–7), 0–30
30s-STS (^§^last contact), repetitions median (IQR), range:				
-Women, *n* = 778	0 (0–0), 0–25	<3	0 (0–0), 0–25	0 (0–5), 0–19
-Men, *n* = 545	0 (0-5), 0–26	0 (0–3), 0–4	0 (0–0), 0–14	0 (0–8), 0–26
Length of stay, days, *n* = 4948^**≠**^	6 (2.4–10.2)	1.0 (0.7–1.9)	4.8 (2.1–8.3)	7.1 (4.0–12.7)

Cumulated Ambulation Score (CAS) is recorded as a 1-day 0–6-point score with 6 points indicating independent basic mobility. 30-s Sit-To-Stand-Test (30s-STS) records the number of stands within 30 s

^*^As questionnaire

^#^initial assessment/first mobilisation attempt

^§^last assessment/upon discharge from hospital

^**≠s**^114 patients died in hospital

## Physical function according to normative reference values

**Fig. 2:**
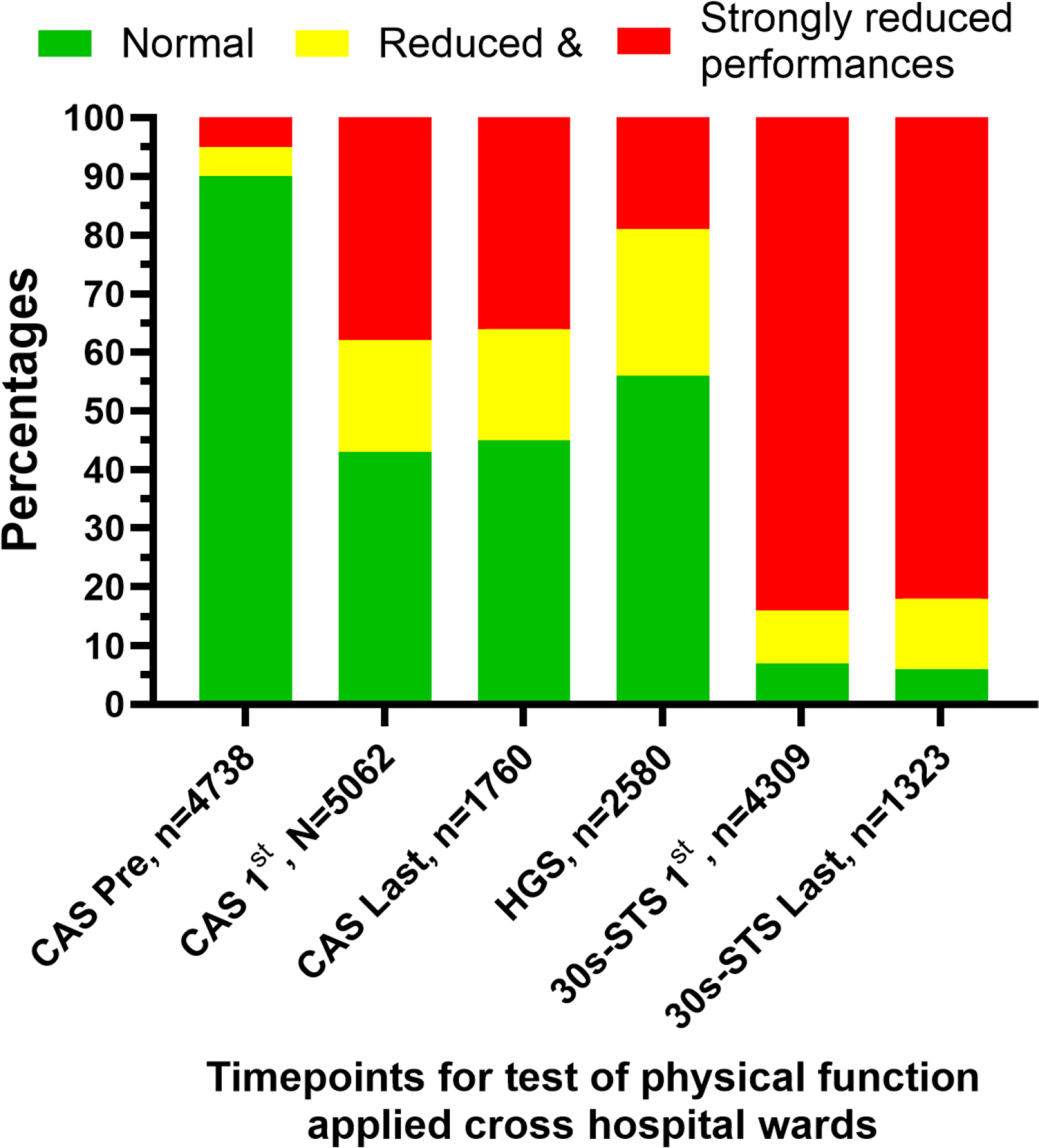
Performances of physical function according to Danish normative reference values for the Cumulated Ambulation Score (CAS), Handgrip strength (HGS) and the 30sec Sit-To-Stand-Test (30s-STS).

Almost all patients (90%) were independent in basic mobility (CAS = 6) at their questionnaire-based pre-admission evaluation. At the corresponding first objective CAS assessment, more than half of patients had a reduced (19%) or strongly reduced (38%) level, while more than 40% of patients had a reduced (25%) or strongly reduced (19%) level of HGS. Further, 84% of patients had a strongly reduced 30s-STS performance at the first assessment (Fig. 2).

## Physical function and 30-day, 90-day, and 1-year mortality

**Fig. 3:**
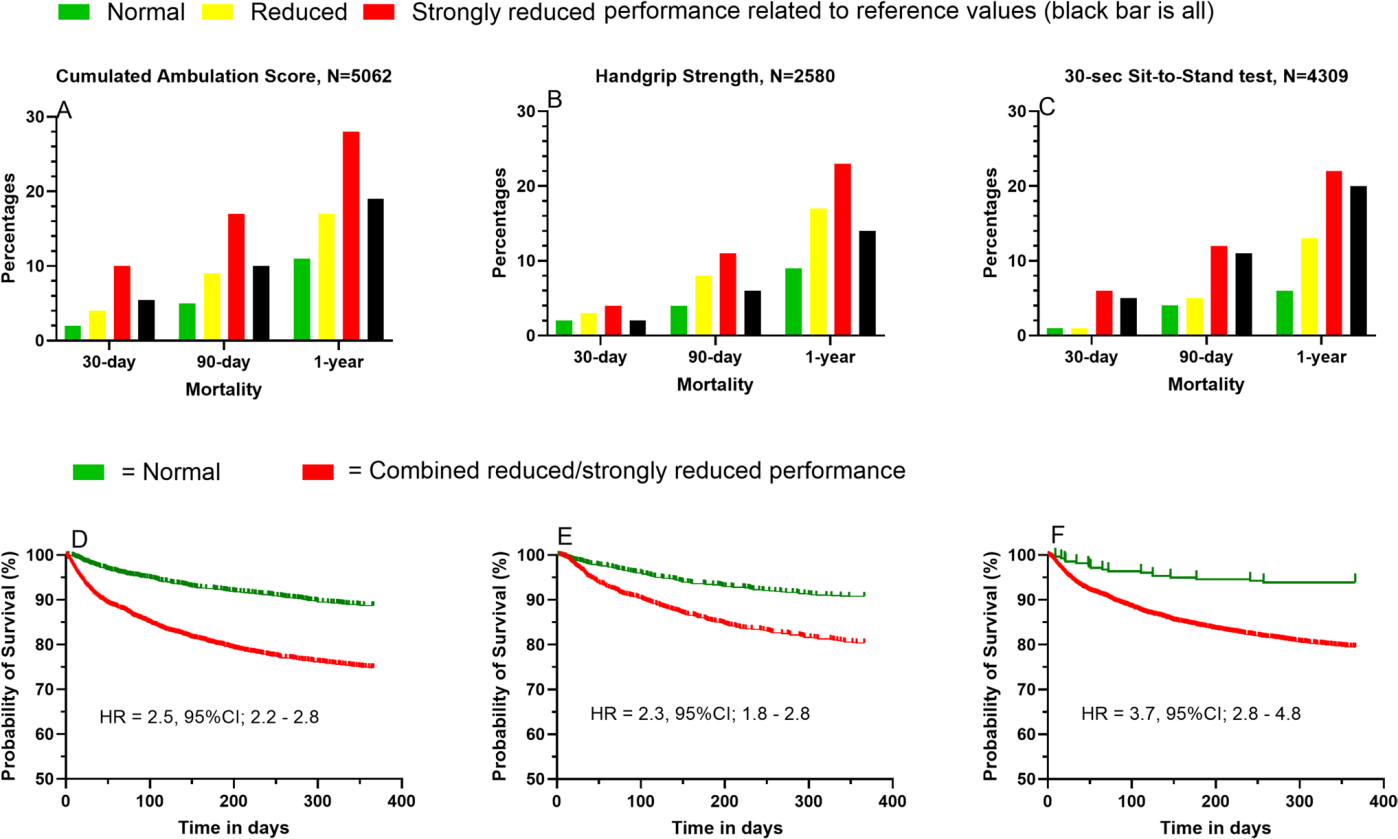
**A**–**F**. Associations between performances of physical function according to the traffic light system and 30-day, 90-day and 1-year mortality (Fig. **A**–**C**), and 1-year Kaplan-Meier survival plots for respective tests (Fig. **D**–**F**)

Overall mortality was 5.4%, 10.4%, and 19.3% at the 30-day, 90-day, and 1-year follow-up, respectively. Reduced/strongly reduced performances of the three tests were associated with increased mortality on all three follow-up timepoints (30-day, 90-day, and 1-year), compared to those with a normal level (Fig. 3A-C, Appendix 3). Corresponding 1-year Kaplan-Meier survival plots illustrate associations (Fig. 3D-F). The 1-year survival rates for patients with a reduced/strongly reduced level for CAS, HGS and 30s-STS level were 59% (HR = 0.41, 95%CI, 0.36–0.46), 56% (HR = 0.44, 95%CI, 0.36–0.54), and 73% (HR = 0.27, 95%CI, 0.21–0.36) lower than in the “normal” group, respectively. The Mantel-Cox log-rank survival distribution tests also differed for CAS = x^2^(1) = 158 (*p* < 0.001), HGS = x^2^(1) = 59 (*p* < 0.001) and for the 30s-STS = x^2^(1) = 32 (*p* < 0.001).

In the adjusted Cox regression models, belonging to the combined reduced/strongly reduced performance category was associated with a significantly increased risk of all-cause 30-day, 90-day, and 1-year mortality by HRs 2.9, 2.3 and 2.0 for the CAS; by 2.2, 2.4 and 2.1 for HGS, and by 3.7, 3.0 and 3.6 for the 30s-STS test versus those with a normal level (Table 2). The 95% CIs for the respective hazard ratios were narrowest for the CAS and followed by the HGS and the 30s-STS test, the latter also being the test with almost all patients having a reduced/strongly reduced performance.

**Table 2:** Multivariable Cox regression analysis of CAS, HGS and 30s-STS performances and mortality at 30-day. 90-day and 1-year

	B	*P*-value	Exp(B); HR	95.0% CI for Exp(B)	
				Lower	Upper
*CAS-first, n = 5062*					
30-day mortality	1.067	<0.001	2.907	2.111	4.003
90-day mortality	.844	<.001	2.326	1.878	2.882
1-year mortality:					
All	.683	<.001	1.980	1.711	2.292
Patients ≥ 85 years, n = 1185	.701	<.001	2.015	1.568	2.590
*Handgrip strength, n = 2580*					
30-day mortality	.801	.003	2.227	1.317	3.765
90-day mortality	.882	<.001	2.416	1.735	3.363
1-year mortality:					
All	.724	<.001	2.062	1.661	2.562
Patients ≥ 85 years, n = 557	.679	<.001	1.972	1.400	2.778
*30s-STS first*					
30-day mortality	1.311	.009	3.711	1.379	9.987
90-day mortality	1.086	<.001	2.961	1.580	5.549
1-year mortality, *n* = 4309					
All	1.273	<.001	3.573	2.208	5.780
Patients ≥ 85 years, 1054	1.201	<.001	3.325	1.570	7.040

The date of first in-hospital CAS assessment was used as index for all analysis. Normal performance (reference) versus reduced/strongly reduced performance was evaluated for alle three tests and adjusted for age, sex and multimorbidity with the M3 index (0–1 vs. >1)

*CAS* Cumulated Ambulation Score, *30s-STS* 30sec Sit-To-Stand test, *HR* hazard ratio

In patients 85 years or older, 1-year mortality was 34 % for reduced CAS (401 out of 1185), 25% for reduced HGS (142 out of 557), and 34% for reduced 30s-STS (355 out of 1054) and with similar HR´s for all three tests as for analysis of the entire cohort (Table 2).

## Differences in patients with and without handgrip strength and sit-to-stand assessments

No significant sex differences were seen for patients with versus without HGS assessments, while those with no HGS assessment were significantly older (mean age, 75.1 (14.0) vs. 73.9 (13.9) years, *p* = 0.02), had more multimorbidity (57.4% vs. 42,6% with M3-index>1, *p*<0.001) and more had a reduced/strongly reduced ambulatory status at their first objective CAS assessment (54.2% vs. 45.8% with CAS<6 points, *p*<0.001) compared to those with HGS assessments. Also, patients without versus with HGS assessments had significantly (*p*<0.001) higher mortality rates at 30 days (8.6% vs. 2.4%), 90 days (14.7% vs. 6.2%), and at 1-year (25.0% vs. 13.8%), indicating that the frailest patients did not have their HGS assessed.

For patients with versus without the first 30s-STS assessment, no significant difference (*p*>0.1) were seen for sex, multimorbidity and mortality (all timepoints). Still, those not being assessed with the 30s-STS were significantly older (mean age, 75.0 (13.5) vs. 71.5 (16.5) years, *p*<0.001), while less had a normal CAS level (41% vs. 54%, *p*<0.001) compared to those with the 30s-STS being assessed.

## Discussion

This prospective cohort study demonstrated that assessment of physical function using simple, validated tests in routine hospital practice provides prognostic information for mortality risk across diverse patient populations.

In 5062 adult patients assessed over one year across hospital wards and different diagnoses, we found that most patients had reduced physical function. Furthermore, impaired physical function was associated with all-cause mortality at the 30-day, 90-day and 1-year following the first in-hospital CAS-assessment. Mortality risks were similar for the CAS, HGS, and the 30s-STS in analysis adjusting for age, sex, and multimorbidity, but with the CAS being associated with the highest mortality rates compared to the other tests at all three timepoints. Similar results were found for patients 85 years or older.

The strong association between the early in-hospital CAS performances of patients and short-term mortality has until now “mainly” been shown in patients with hip fracture–where the score was developed and first validated [36]. Since then, the use of the CAS has expanded to, i.e. patients undergoing acute high-risk abdominal surgery [24], geriatric patients [37], patients with vertebral fractures [22], and stroke patients [38]. Moreover, the strong association between reduced CAS and increased mortality, with nearly doubled 1-year mortality risk emphasizes the importance of basic mobility assessment for survival outcomes. The use of CAS in patients across wards and diagnoses at the hospital level in the present study underlines the importance of patients having an independent ambulatory status (CAS = 6). At the same time, it clearly shows that the CAS can be considered a generic measure that can be used for the initial evaluation of all patients, identifying those at highest risk for a fatal outcome, both at shorter and longer time periods. The latter adds to the value of using the CAS in hospital settings and the importance of an immediate enhanced focus for patients with a reduced or strongly reduced level. Correspondingly, an independent ambulatory CAS-status is considered an important first-step goal for the early rehabilitation of patients, regardless of age and diagnosis. Preferably, this goal is achieved for most patients before being discharged, as this has been associated with increased survival and reduced re-admission in i.e., patients with hip fracture [12]. If not reached, this goal should be given a very high attention from the very first day after discharge in municipalities.

Despite implementation of the systematic assessment, and HGS being the test with the fewest assessments (only about half of patients) there was a significant and robust association with mortality at all three timepoints. To be noticed, patients not being assessed with HGS were older, had more multimorbidity, had a lower ambulatory status, and more died compared to patients with HGS assessment performed. Nevertheless, according to a previous study implementing HGS in five medical wards, about 80% of patients were able to perform the test [39, 40], providing clear indications of more patients that could have been assessed in the present study. Still, although more patients could have been assessed, our findings of reduced HGS being associated with an increased risk of mortality are supported by studies of different patient groups [5, 41] and both younger and older adults [3, 6, 42, 43]. Thus, supporting the suggestion of HGS as an overall “new vital sign of health” [26].

We found a clear association between the 30s-STS test and an increased risk of mortality over one year for those with a reduced/strongly reduced performance compared to the few (<10%) with a normal level for the 30s-STS test. With less than 10% of patients being evaluated with the 30s-STS having a normal performance, any conclusions can be difficult. In comparison, performances of the STS test in community-dwelling people have been associated with mortality at 4–10 years follow-up [3].

Importantly, one purpose of including the 30s-STS test in this test battery is that all patients achieve a result, no matter if they cannot even stand up a single time, equal to a result of zero repetitions. Thus, it holds the potential to monitor small but important improvements in performance, i.e., from 0 to 3 repetitions, and for continued monitoring of progress in further rehabilitation provided after discharge from the acute hospital. In comparison, the 5-times STS test requires that a person perform a minimum of 5 repetitions to get a result.

A strength is the prospective cohort design, with planned, systematic assessment of adult patients across all hospital wards. The design eliminates selection bias associated with retrospective studies providing a robust foundation for examining exposure-outcome relationships. The innovative integration of standardized testing protocols with electronic health records, including automated algorithms and traffic light visualization, demonstrates how systematic data collection can be embedded into clinical workflows. Prospective enrollment of 5062 patients over a one year with one-year mortality follow-up data provides a large study sample and shows sustained prognostic value beyond hospitalization. The comprehensive test battery assessing mobility (CAS), strength (HGS), and function (30s-STS) provides a comprehensive evaluation superior to single-test approaches, while implementation within routine clinical practice enhances external validity and shows feasibility for other hospital settings.

Some limitations also need attention. Gaps in data collection occurred, particularly for HGS (51% assessed) compared to CAS (98%) and 30s-STS (85%). Patients not assessed for HGS were systematically different, being older with greater multimorbidity and higher mortality rates, introducing selection bias that may underestimate the true association between reduced grip strength and mortality. So, we choose not to include all three tests in the same analysis, to evaluate if one where superior to the other tests, as this would have been on a markedly reduced and different population. Integration of HGS into routine clinical workflows meant assessment completion was subject to clinical priorities and staffing levels, compromising data completeness compared to controlled research settings. For HGS, this meant that the recommendation now is that it should be assessed, if possible, during the first session where CAS and 30s-STS tests are objectively assessed. The 30s-STS showed poor discriminative ability, with over 90% of patients showing reduced performance, severely limiting its clinical utility, and raising questions about reference value appropriateness for hospitalized patients. Still, with the ambition of the test-battery being used across sectors, these results provide strong indications of patients with a high need for further rehabilitation following discharge from the hospital, and with progress monitored with the 30s-STS for those who have reached an independent CAS-level. Another limitation is that data on other known factors predictive of mortality, i.e., frailty-, nutritional and cognitive status were not recorded along with the three physical function tests, which would have provided the opportunity also to account for these factors in our multivariable analysis. Still, dementia and malnutrition were among the many chronic conditions that defined the M3-index, and thereby to some extent considered.

Despite the prospective design, the observational nature precludes definitive causal conclusions about whether reduced physical function directly contributes to mortality or serves as a marker of underlying disease severity. Still, identifying high-risk patients provides the opportunity for clinicians to instigate a multidisciplinary enhanced recovery program, including nutrition and physical exercise focused on improving physical function, and thereby potentially improve other outcomes. Thus, a more intensive physiotherapy program with two daily sessions on weekdays versus usual care improved the in-hospital basic mobility level [44], and with increased survival rates seen for patients recovering their basic mobility at discharge from acute hospitals after surgery for hip fracture [12]. Others have shown similar positive associations related to the extent of physiotherapy (the more the better) within the 1^st^ week after surgery for hip fracture [45]. On the other hand, some patients might be severely ill, where better mobility might not change their estimated lifetime.

Future large-scale research studies using physical function test results to stratify patients to a more intensive acute hospital exercise program are needed to evaluate the effect on the early ambulatory status and thereby potentially improve short- and long-term outcomes. Where necessary, such intensive programs should be continued immediately after discharge in the municipality.

## Conclusions

In unselected medical and surgical older patients, 30-day, 90-day, and 1-year mortality markedly increase with reduced or strongly reduced physical function identified by three simple and non-time-consuming tests. Assessment of CAS, HGS, and 30s-STS can easily be implemented as a generic core test battery of physical function in routine clinical practice in other hospitals and across sectors.

## Supplementary Information

Below is the link to the electronic supplementary material.

Supplementary file1 (DOCX 133 KB)
