# Supplementary material for: Assessment of physical function of hospitalized older patients in routine clinical practice predicts 1-year mortality; A cohort study of 5,062 medical and surgical patients
Source: Eur Geriatr Med. 2026 Feb 28;17(3):1467–78. doi: 10.1007/s41999-026-01439-5 (PMC13309418; doi:10.1007/s41999-026-01439-5)
Supplement: Supplementary file 1 — Supplementary file1 (DOCX 133 KB) [file 41999_2026_1439_MOESM1_ESM.docx]

**Appendix 1**

| **Danish reference values for Cumulated Ambulation Score (CAS) - Handgrip strength (HGS) & 30s Sit-To-Stand test (30s-STS)** | | | | | | | | | |
| --- | --- | --- | --- | --- | --- | --- | --- | --- | --- |
|  | **CAS, 0-6 points** | | | **HGS, Kg** | | | **30s-STS, repetitions** | | |
| **Age. years** | **Normal** | **Reduced** | **Strongly**  **reduced** | **Normal** | **Reduced** | **Strongly**  **reduced** | **Normal** | **Reduced** | **Strongly**  **reduced** |
| ***Women*** | | | | | | | | | |
| **18-29** | **6** | **4-5** | **< 4** | **26.8 – 38.6** | **20.9 – 26.7** | **< 20.9** | **19 - 30** | **14 - 18** | **< 14** |
| **30-39** | **6** | **4-5** | **< 4** | **27.8 – 39.4** | **22.0 – 27.7** | **< 22.0** | **19 - 30** | **13 - 18** | **< 13** |
| **40-49** | **6** | **4-5** | **< 4** | **27.4 – 37.6** | **22.3 – 27.3** | **< 22.3** | **17 - 28** | **11 - 16** | **< 11** |
| **50-59** | **6** | **4-5** | **< 4** | **24.7 – 35.1** | **19.5 – 24.6** | **< 19.5** | **15 - 26** | **9 - 14** | **< 9** |
| **60-69** | **6** | **4-5** | **< 4** | **21.8 – 31.6** | **16.9 – 21.7** | **< 16.9** | **13 - 23** | **8 - 12** | **< 8** |
| **70-79** | **6** | **4-5** | **< 4** | **19.2 – 28.2** | **14.7 – 19.1** | **< 14.7** | **11 - 19** | **7 - 10** | **< 7** |
| **80-89** | **6** | **4-5** | **< 4** | **16.2 – 24.4** | **12.1 – 16.1** | **< 12.1** | **10 - 17** | **6 - 9** | **< 6** |
| **≥ 90** | **6** | **4-5** | **< 4** | **12.4 – 18.6** | **9.3 – 12.3** | **< 9.3** |  |  |  |
| ***Men*** | | | | | | | | | |
| **18-29** | **6** | **4-5** | **< 4** | **42.3 – 58.1** | **34.4 – 42.2** | **< 34.4** | **19 - 30** | **14 - 18** | **< 14** |
| **30-39** | **6** | **4-5** | **< 4** | **44.5 – 60.5** | **36.5 – 44.4** | **< 36.5** | **20 - 31** | **15 - 19** | **< 15** |
| **40-49** | **6** | **4-5** | **< 4** | **44.7 – 59.7** | **37.2 – 44.6** | **< 37.2** | **19 - 29** | **14 - 18** | **< 14** |
| **50-59** | **6** | **4-5** | **< 4** | **41.6 – 55.4** | **34.7 – 41.5** | **< 34.7** | **16 - 27** | **11 - 15** | **< 11** |
| **60-69** | **6** | **4-5** | **< 4** | **37.1 – 51.9** | **29.7 – 37.0** | **< 29.7** | **13 - 24** | **8 - 12** | **< 8** |
| **70-79** | **6** | **4-5** | **< 4** | **31.2 – 45.6** | **24.0 – 31.1** | **< 24.0** | **12 - 21** | **7 - 11** | **< 7** |
| **80-89** | **6** | **4-5** | **< 4** | **25.5 – 38.9** | **18.8 – 25.4** | **< 18.8** | **9 - 18** | **5 - 8** | **< 5** |
| **≥ 90** | **6** | **4-5** | **< 4** | **14.7 – 27.3** | **8.4 – 14.6** | **< 8.4** |  |  |  |
| Green = normal level (based on mean ± 1SD for HGS og 30s-STS decades). Yellow = Reduced level (interval between normal and strongly reduced) Red = Strongly reduced level (< -2SD). Colors for CAS are based on consensus and evidence. Kristensen MT. Dall CH. Aadahl M. Suetta C. [Systematic assessment of physical function in adult patients across diagnoses.](https://pubmed.ncbi.nlm.nih.gov/36331169/) Ugeskr Laeger. 2022 Oct 24;184(43) | | | | | | | | | |

**Appendix 2**

**Appendix 3**

| Association between tests of physical function and 30-day, 90-day and 1-year mortality in patients assessed in acute, surgical and medical wards. | | | | |
| --- | --- | --- | --- | --- |
| Exposure variables | **N = 5062** | **Mortality** | | |
|  |  | **30-day** | **90-day** | **1-year** |
| CAS first*, n=5062: | | | | |
| Normal, 6 points | 2172 (43) | 46 (2.1) | 108 (5.0) | 247 (11.4) |
| Reduced, 4-5 points | 974 (19) | 40 (4.1) | 89 (9.1) | 188 (19.3) |
| Strongly reduced, 0-3 points | 1916 (38) | 189 (9.9) | 327 (17.1) | 542 (28.3) |
| Total mortality |  | 275 (5.4) | 524 (10.4) | 977 (19.3) |
| Handgrip Strength*, n=2580: | | | | |
| Normal | 1433 (56) | 22 (1.5) | 54 (3.8) | 132 (9.2) |
| Reduced | 650 (25) | 20 (3.1) | 51 (7.8) | 113 (17.4) |
| Strongly reduced | 497 (19) | 20 (4.0) | 55 (11.1) | 112 (22.5) |
| Total mortality |  | 62 (2.4) | 160 (6.2) | 357 (13.8) |
| 30s-STS first*, n=4309 | | | | |
| Normal | 279 (7) | 4 (1.4) | 10 (3.6) | 17 (6.1) |
| Reduced | 404 (9) | 4 (1.0) | 18 (4.5) | 51 (12.6) |
| Strongly reduced | 3626 (84) | 225 (6.2) | 427 (11.8) | 780 (21.5) |
| Total mortality |  | 233 (5.4) | 455 (10.6) | 848 (19.7) |
| Data are numbers (percentages). CAS; Cumulated Ambulation Score (0-6 points), 30s-STS; 30sec Sit-To-Stand test.  *Mortality for all comparisons is related to the date of first objective CAS assessment in the hospital  *Normal, reduced and strongly reduced are related to normative Age-decade and sex related levels. “First” indicate the first objective assessment after referral to Physical or Occupational therapy for the evaluation of the functional level | | | | |


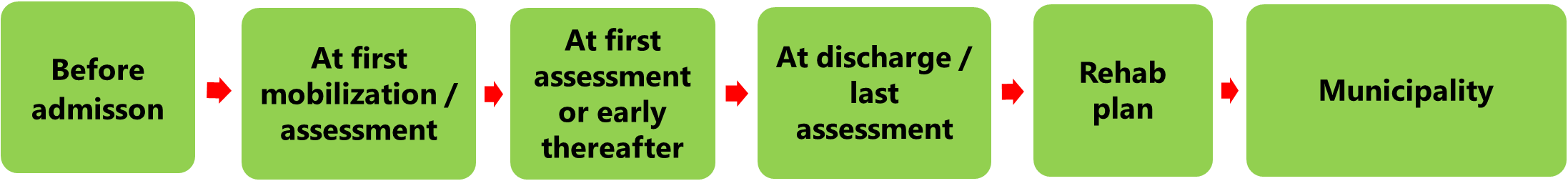


**CAS & STS last**

**CAS, HGS & STS**

**HGS**

**CAS & STS 1st**

**CAS Pre**

**Timeline for test of physical function with CAS, HGS and 30s-STS**

**CAS - Cumulated Ambulation Score: Basic mobility**

**HGS - Handgrip strength: Proxy for general strength**

**STS - 30sec–Sit-To-Stand test: Function & strength in lower extremities**
